# Mucosal Healing of Gastric Antral Vascular Ectasia (Watermelon Stomach) After Treatment With Azathioprine

**DOI:** 10.7759/cureus.70490

**Published:** 2024-09-30

**Authors:** George Stancu, Elena Laura Iliescu

**Affiliations:** 1 Gastroenterology and Hepatology, Valahia Medical Center, Ploiesti, ROU; 2 Department of Internal Medicine, Fundeni Clinical Institute, Bucharest, ROU; 3 Internal Medicine, Carol Davila University of Medicine and Pharmacy, Bucharest, ROU; 4 Department of Internal Medicine II, Fundeni Clinical Institute, Bucharest, ROU

**Keywords:** anemia, chronic gastric bleeding, chronic gastrointestinal bleeding, gastric antral venous ectasia, gastric mucosal hemorrhages, gave, management of gastric bleeding, upper gastro-intestinal bleed, watermelon stomach, watermelon stomach treatment

## Abstract

Gastric antral vascular ectasia (GAVE), commonly known as “watermelon stomach,” is characterized by parallel red stripes resembling watermelon stripes on endoscopic examination and is an uncommon but significant cause of chronic gastrointestinal bleeding, often associated with systemic diseases such as autoimmune conditions, liver cirrhosis, chronic renal insufficiency, and cardiovascular disease. Various therapeutic approaches have been introduced for GAVE treatment, including medical, endoscopic, and surgical interventions.

We report a case of a 60-year-old man with a prior history of GAVE who developed melena and symptomatic severe anemia. Initial esophagogastroduodenoscopy (EGD) demonstrated oozing antral GAVE. The patient required weekly blood transfusions. In this article, we explored the safety and efficacy of azathioprine treatment for GAVE. We administered 100 mg (oral) of azathioprine once daily and evaluated the patient monthly for four months. After two months, the endoscopy examination results showed visible macroscopic improvement, and after four months, the lesions were healed. The healing process was complete with normal mucosa restored. After two months, there wasn't any need for blood transfusions or iron therapy. We present for the first time the endoscopic healing process of GAVE under azathioprine treatment confirming that the cause of GAVE is autoimmune. Further research is needed to optimize therapeutic strategies and improve patient outcomes.

## Introduction

Gastric antral vascular ectasia (GAVE), also known as "watermelon stomach," is a rare condition characterized by the development of dilated blood vessels in the stomach's antrum. This condition can lead to chronic gastrointestinal bleeding, anemia, and other complications. Traditionally, various treatments have been employed to manage this disorder, including endoscopic therapies, pharmacological interventions, and surgical approaches.

Recent evidence suggests that the immunosuppressant drug azathioprine may be a promising treatment option for patients with this condition [1]. Gastric antral vascular ectasia is often associated with underlying systemic diseases, such as autoimmune disorders, liver cirrhosis, and chronic renal failure [2]. The exact pathogenesis of this condition remains unclear, but it is thought to involve vascular dysfunction, mucosal ischemia, and abnormal angiogenesis [1].

Endoscopically, the characteristic appearance of longitudinal rugal folds with visible, convoluted blood vessels converging on the pylorus is considered diagnostic for this condition [3]. Histological analysis typically reveals dilated mucosal capillaries, thrombosis, and fibromuscular hyperplasia of the lamina propria. While various treatment modalities have been employed, the management of gastric antral vascular ectasia remains challenging. Since the etiologic mechanism of GAVE is unknown and autoimmunity can be a possible mechanism, we tried to treat a patient with azathioprine .

## Case presentation

**Figure 1:**
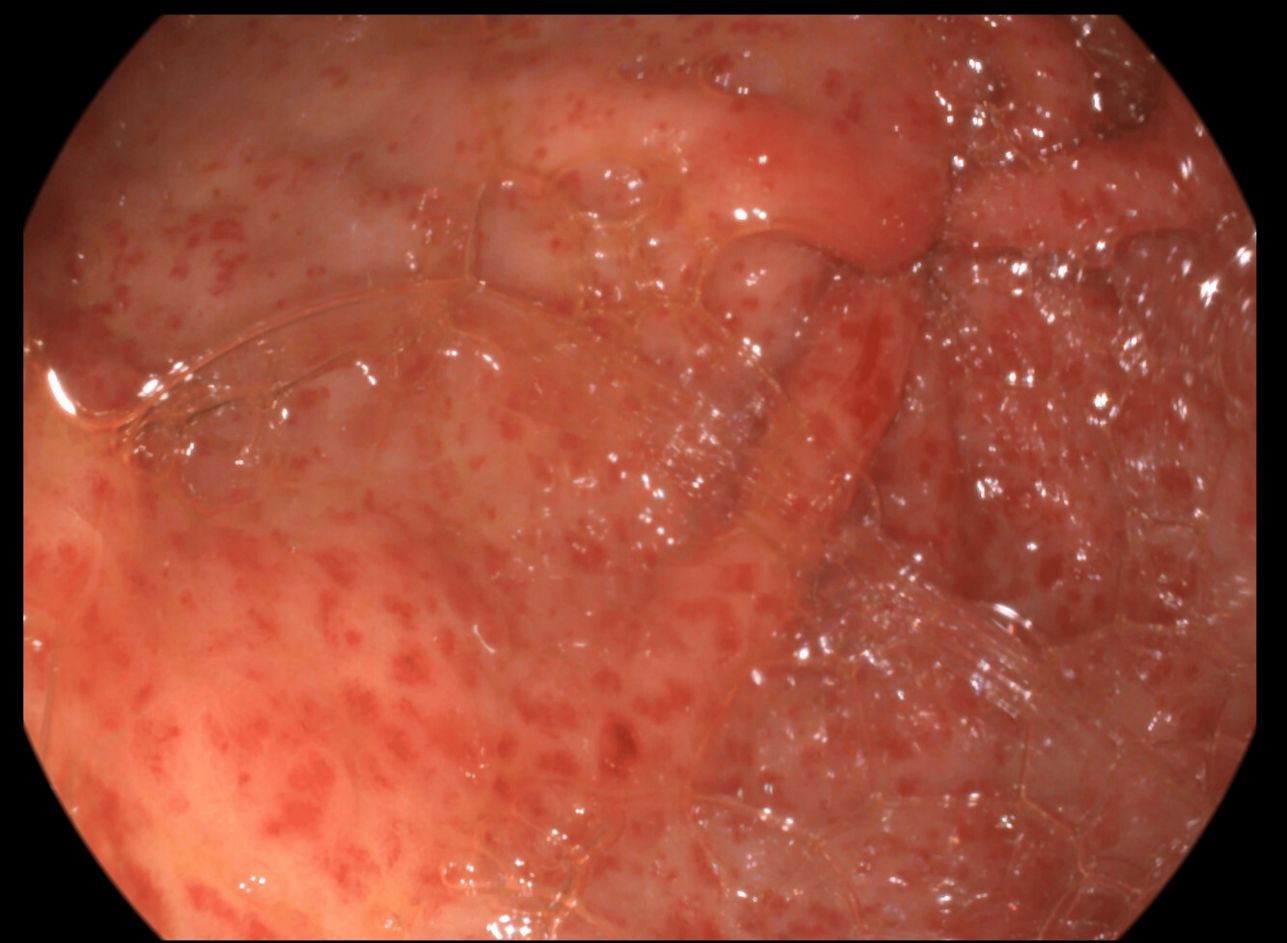
Endoscopic view of the gastric antral region at week 0 The image demonstrates the presence of GAVE (gastric antral vascular ectasia) lesions.

This is the case of a 60-year-old white caucasian male diagnosed with alcoholic liver cirrhosis for five years. The patient developed severe iron deficiency anemia six months ago, and he was treated in another county hospital with repeated blood transfusions, fitomenadione, and proton pump inhibitors. Despite the treatment received, the patient was admitted into the gastroenterology ward for recurrence of severe anemia secondary to upper digestive bleeding every 10 days. He was referred to our University Hospital, Fundeni Clinical Institute, Bucharest, in December 2023. Our evaluation included upper endoscopic examination that revealed gastric antral vascular ectasia (GAVE) (Figure 1) as the source of the chronic bleeding.

The treatment plan for this patient included the correction of severe anemia and the treatment of the bleeding gastric source. The standard treatment for GAVE in our ward is Argon plasma coagulation of gastric lesions. With the usual treatment with endoscopic Argon plasma coagulation for GAVE, we usually obtain a sustained reduction in the bleeding severity for six months. After that, the patient usually develops a recurrence of severe anemia. 

The etiology of GAVE is not clearly known at this moment, and there are papers that suggest that could be an autoimmune mechanism. We decided to start immunosuppression for this patient after the informed consent was obtained. Our primary objective was to determine if immunosuppression can be used to treat GAVE and the secondary objective was to determine the time necessary to obtain gastric mucosal healing. The patient was evaluated for any infectious risk that can be amplified by immunosuppression. Tests for inflammation and serum procalcitonin, QuantiFERON blood test, viral hepatitis and HIV were negative.

The treatment with azathioprine 50 mg tablets twice daily was initiated, and the patient was monitored with clinical examinations, laboratory blood tests, patient history, and digestive endoscopy every four weeks for four months. The variables monitored for this patient were sex, age, ethnicity, BMI, blood transfusion history, the presence of melena, haemoglobin, haematocrit, upper digestive endoscopy imaging, medication, and survival. The pictures obtained from the digestive endoscopy examinations were interpreted by two senior gastroenterology physicians. All the variables are presented in Table 1.

**Table 1:** Patient variables evolution Haemoglobin normal values (13.2 to 18.0 g/dL ); Hematocrit Normal values (41% to 50%); tb: Tablet; BID: Twice a day.

Item	Week 0	Week 4	Week 8	Week 12	Week 16
Recent upper intestinal bleeding	Yes (melena)	Yes (melena)	No	No	No
Recent blood transfusion	Yes	Yes	No	No	No
Haemoglobin	5.9 g/dL	7.3 g/dL	10 g/dL	14.9 g/dL	14.8 g/dL
Hematocrit	19 %	25%	31%	42.3%	42.8%
Endoscopy	Yes	Yes	Yes	Yes	Yes
Treatment	Azathioprine tb 50 mg BID	Azathioprine tb 50 mg BID	Azathioprine tb 50 mg BID	Azathioprine tb 50 mg BID	Azathioprine tb 50 mg BID

**Figure 2:**
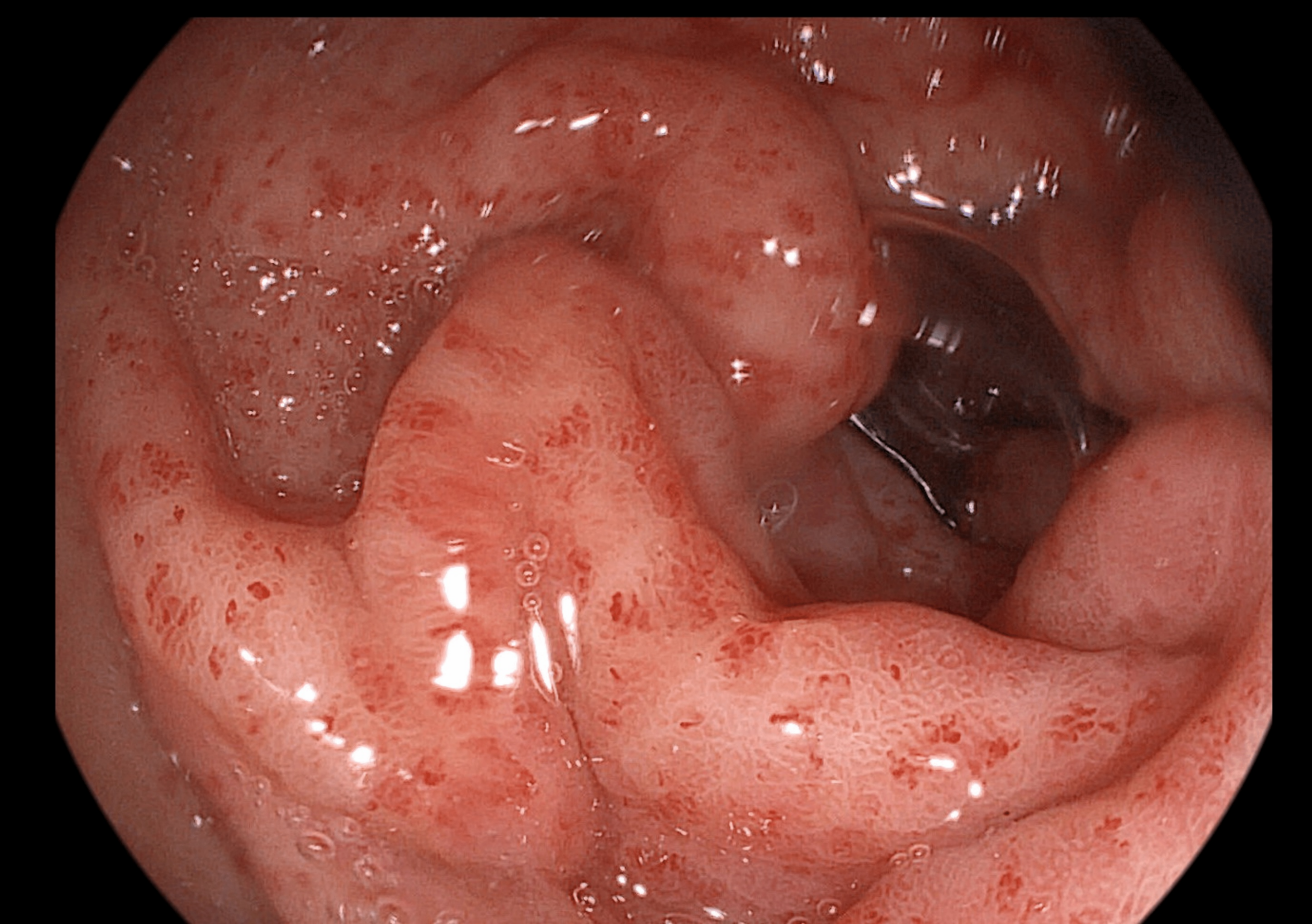
Endoscopic view of the gastric antral region after two weeks of treatment The lesions are similar to the week 0 examination.

After two weeks of treatment, the patient returned to the hospital because the melena persisted. We had to perform an unscheduled upper digestive endoscopy to exclude another concomitant cause of bleeding that could have appeared since the last examination. The aspect of the antral region was similar to that of the week 0 examination (Figure 2).

**Figure 3:**
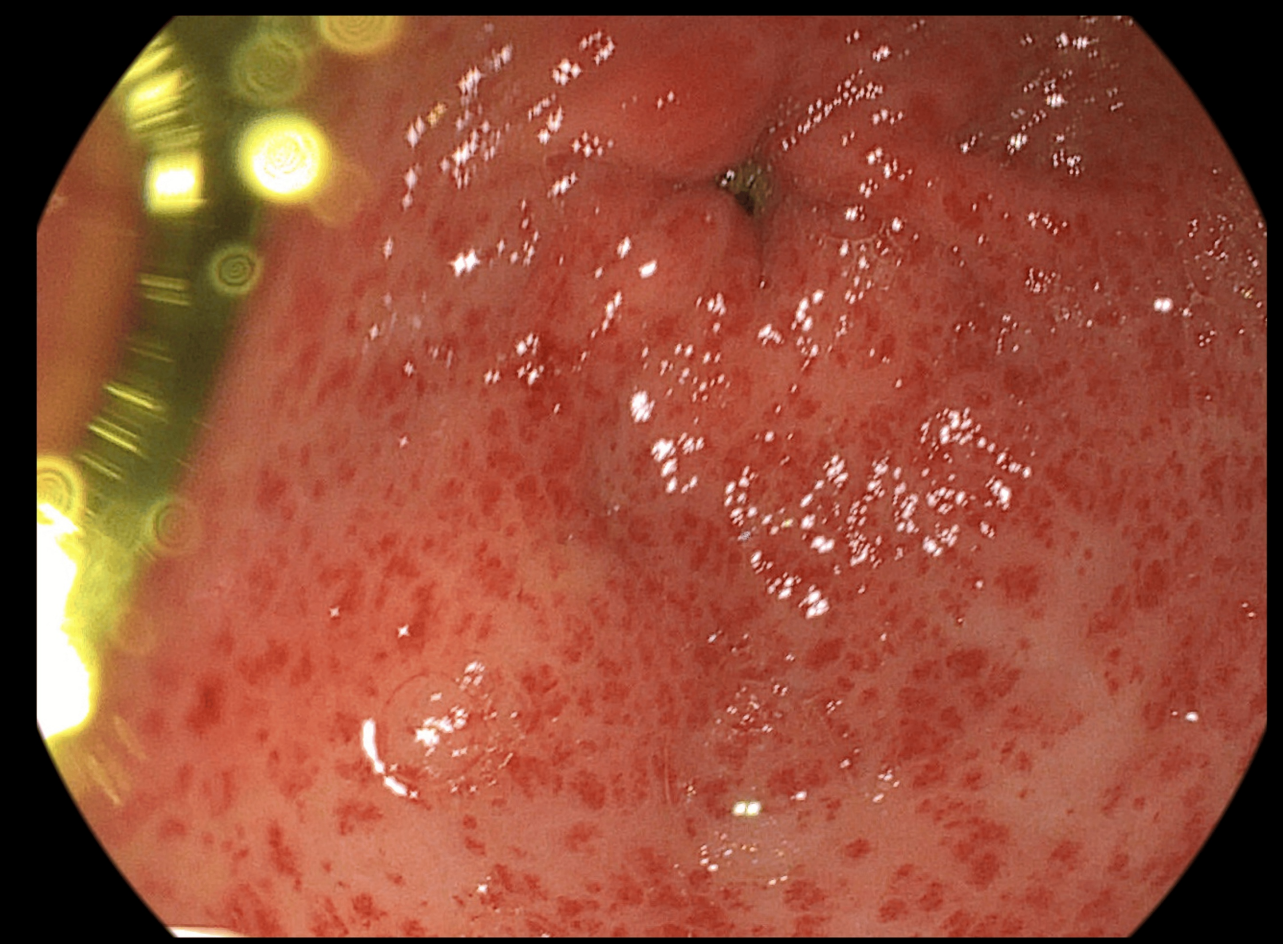
Endoscopic view of gastric antral region at week 4 (one month of treatment with azathioprine)

After one month, an upper digestive endoscopy was performed, and the antral mucosa showed the same severity as GAVE (Figure 3). The patient was advised to continue the treatment with azathioprine using the same dose.

**Figure 4:**
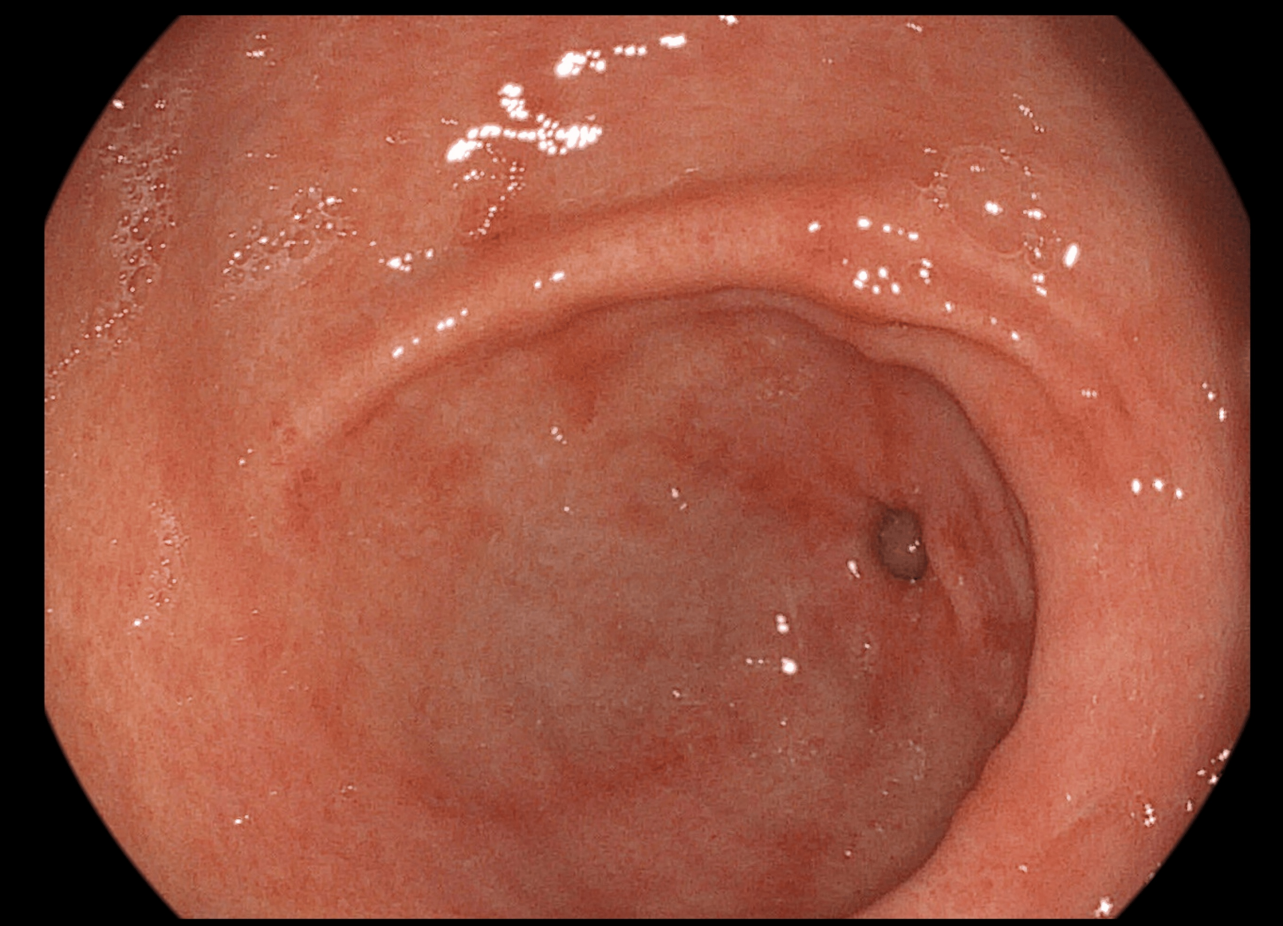
Endoscopic view of the gastric antral region at the week 8 evaluation

At the end of the second month of treatment with azathioprine, at the week 8 evaluation, the patient reported that the melena wasn't present. The last episode of melena was observed around the sixth week of treatment. The patient also had an increase in the blood haemoglobin level. The patient reported that the last blood transfusion was received on week 6. The endoscopic examination after eight weeks of treatment revealed that the antral lesions of GAVE are significantly improved (Figure 4). 

**Figure 5:**
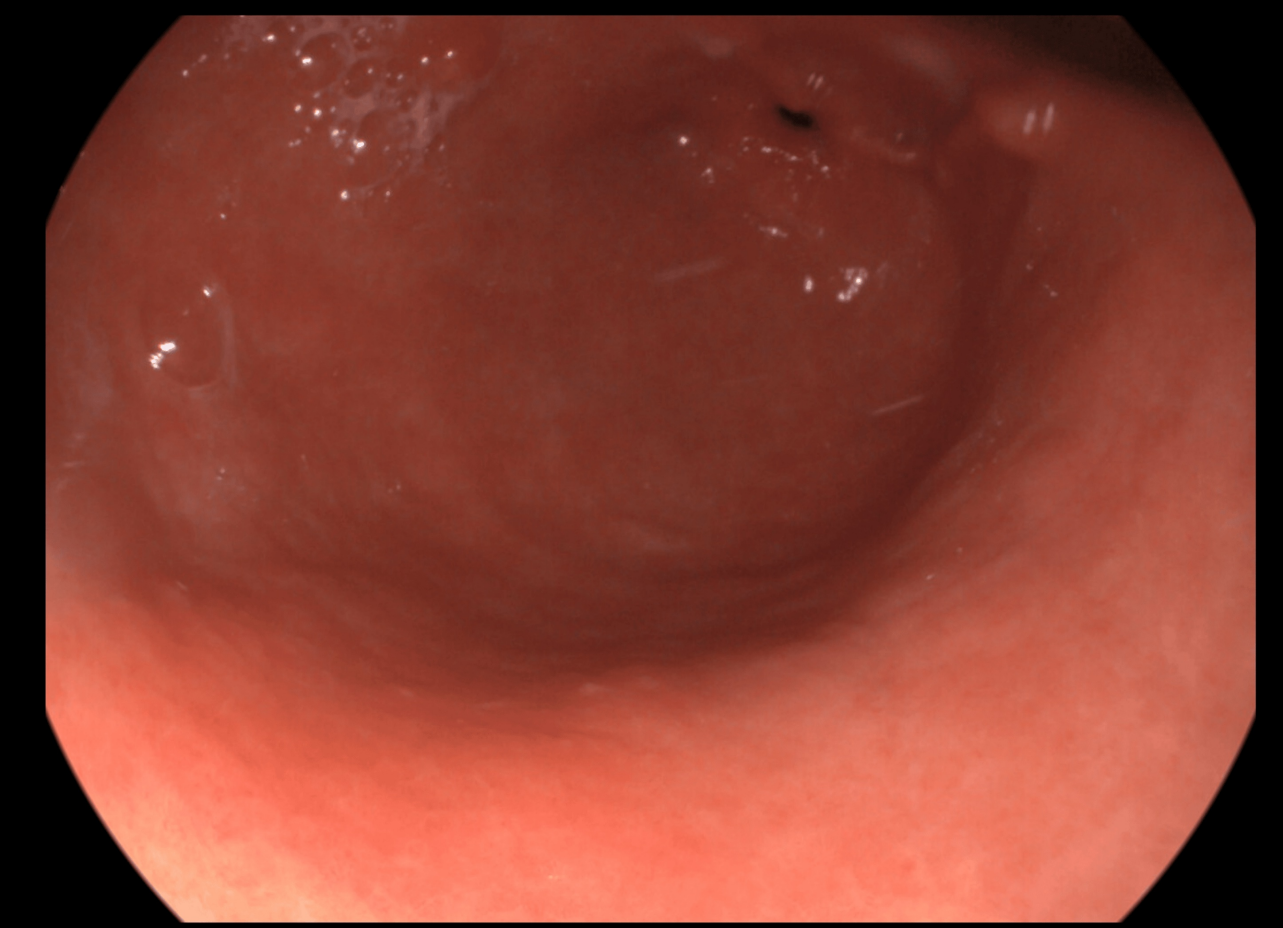
Endoscopic view of gastric antral region at week 12 examination.

**Figure 6:**
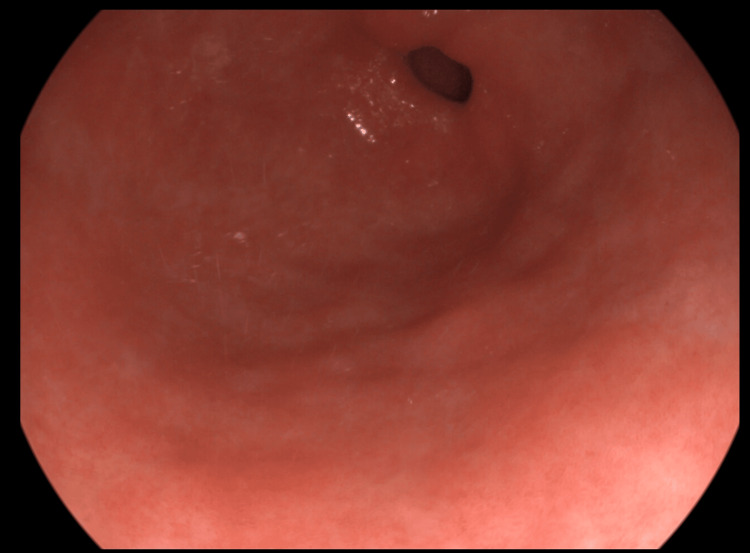
Endoscopic view of gastric antral region at week 16 examination.

The patient was advised to continue the treatment. At the week 12 evaluation, after three months of treatment, the clinical, biological, and endoscopic variables were almost normal (Figure 5), and this status was maintained until the fourth month of treatment (week 16) with azathioprine (Figure 6). 

All the images were obtained with a Pentax Defina Endoscope (PENTAX Medical, Japan).

## Discussion

GAVE is a condition that induces chronic severe anaemia by continuous oozing bleeding from gastral antral mucosal lesions [4]. The macroscopic aspect of these lesions is characteristic: longitudinal rugal folds traversing the antrum and converging on the pylorus [5]. This aspect is similar to the stripes of a watermelon [1,2]. The histopathological abnormalities of these stripes consist of anomalies in small vessels and capillaries [1,2], such as dilatation of mucosal capillaries with focal thrombosis and fibromuscular hyperplasia of the lamina propria. In resected specimens was found thickened mucosa with tortuous submucosal venous vessels [2,5].

The GAVE lesions can appear in a short period of time [2]. Our patient was diagnosed with anaemia about two months ago. Since he was known to have cirrhosis, portal hypertension complications were the usual suspects, but the first endoscopic examination ruled out oesophageal varix and portal gastropathy. The persistence of melena and the fast recurrence of anaemia raised the suspicion of GAVE and the upper digestive endoscopy and mucosal biopsy confirmed it.

The current treatment options for GAVE includes medical, endoscopic and surgery. Correction of anaemia with recurrent blood transfusions and iron repletion therapy could maintain the blood levels. Endoscopic treatment with radiofrequency ablation for gastric antral vascular ectasia involves using radiofrequency (heat) energy to stop the bleeding by destroying the enlarged blood vessels. Also Plasma Argon Coagulation (APC) and endoscopic band ligation (EBL) will temporary induce a temporary control over the bleeding. With all these technics we obtain only a temporary effect. The bleeding will resume after several weeks [6]. There are no medical treatment recommendations for GAVE so an immunosuppressive treatment can be an useful option [7].

After the initiation of the azathioprine treatment, the clinical evolution of our patient wasn’t changed for the first month of treatment. In the second month of treatment, the patient observed the resolution of melena and the value of haemoglobin kept rising (Table 1). After the second month the patient has non need for blood transfusion or iron therapy and the need for hospitalisation decreased from weekly to once monthly.

The most spectacular change was observed in the mucosal aspect of gastric antral mucosa. The number of vascular lesions didn’t decrease in the first month (Figures 1-3), but after week 4, the healing process was macroscopically visible with every digestive endoscopy examination (Figures 4-6]. By the week 8 endoscopic examination there was no visible bleeding from the antral lesions (Figure 4). After that the endoscopic examinations from week 12 and 16 showed a normal mucosa without any vascular lesions.

The treatment with azathioprine induced mucosal healing of gastric antral vascular lesion and the primary objectif of this case was obtained. We proved that mucosal healing in GAVE can be obtained with medical immunosuppressive treatment. The second objective was to determine the time needed to obtain mucosal healing, which, for this patient, was eight to 12 weeks. 

## Conclusions

Gastric antral venous ectasia represents a serious and possible life-threatening by inducing severe anaemia. The etiology of the vascular alterations in GAVE remains unknown. The current treatment options (steroids, surgery, endoscopic plasma argon coagulation, endoscopic band ligation, Neodymium-doped yttrium aluminium garnet laser coagulation, cryotherapy, and radiofrequency ablation) provide only a temporary amelioration of symptoms. The persistence of GAVE induce iron deficiency anaemia that needs to be treated with blood transfusions and iron repletion therapy. These patients need to be closely monitored for relapse of anaemia.

A medical treatment that induces complete healing of the gastric antral mucosa after four months of treatment with an immunosuppressive drug, azathioprine, will increase the quality of life and life expectancy of these patients and reduce the treatment and hospitalisation costs. Azathioprine induced mucosal healing in four months in one patient, but further research is needed to optimize therapeutic strategies and improve patient outcomes.

## References

[1] Watson M, Hally RJ, McCue PA, Varga J, Jiménez SA (1996). Gastric antral vascular ectasia (watermelon stomach) in patients with systemic sclerosis. Arthritis Rheum.

[2] Hsu WH, Wang YK, Hsieh MS (2018). Insights into the management of gastric antral vascular ectasia (watermelon stomach). Therap Adv Gastroenterol.

[3] Jabbari M, Cherry R, Lough JO, Daly DS, Kinnear DG, Goresky CA (1984). Gastric antral vascular ectasia: the watermelon stomach. Gastroenterology.

[4] Gostout CJ, Viggiano TR, Ahlquist DA, Wang KK, Larson MV, Balm R (1992). The clinical and endoscopic spectrum of the watermelon stomach. J Clin Gastroenterol.

[5] Novitsky YW, Kercher KW, Czerniach DR, Litwin DE (2003). Watermelon stomach: pathophysiology, diagnosis, and management. J Gastrointest Surg.

[6] Abdo M, Moustafa A, Mostafa I (2022). To coagulate, ligate, or both: a randomized study comparing the safety and efficacy of two endoscopic approaches for managing gastric antral vascular ectasia in cirrhotic patients. Egypt Liver Journal.

[7] Lowes JR, Rode J (1989). Neuroendocrine cell proliferations in gastric antral vascular ectasia. Gastroenterology.

